# Enhancing antioxidant activity of corn bract and silk juices through biotransformation of polyphenols by *Lactobacillus paracasei* TJ199 fermentation

**DOI:** 10.1016/j.fochx.2025.102586

**Published:** 2025-05-26

**Authors:** Xiaohui Tang, Huanyong Lv, Hailong Wang, Huixin Yang, Baogang Zhang, Ling Cong, Xinyi Li, Menghan Ma, Yunhe Xu, Lili Zhang

**Affiliations:** a Liaoning Provincial Professional Technology Innovation Center of Meat Processing and Quality-Safety Control, College of Food and Health, Jinzhou Medical University, Jinzhou 121000, China; b Liaoning Agricultural Vocational and Technical College, Yingkou 115007, China; c Institute of Scientific and Technological Development, Shenyang Agricultural University, Shenyang 110161, China; d Comparative Molecular Biosciences Graduate Program, University of Minnesota–Twin Cities, St. Paul, MN, 55108 Paul, MN, United States; e College of Pharmacy, Jinzhou Medical University, Jinzhou 121001, China; f The Third Affiliated Hospital of Jinzhou Medical University, Jinzhou 121001, China

**Keywords:** Corn bract juice, Corn silk juice, Widely targeted metabolomic analysis, Phenolic compounds, Antioxidant activity, Lactic acid fermentation

## Abstract

To enhance the bioactivity of corn bract and silk, we isolated *Lactobacillus paracasei* TJ199 - a strain with exceptional antioxidant capacity - was used to ferment corn bract juice (CBJ) and silk juice (CSJ). Fermentation increased total phenolic (0.47 - and 0.23 - fold) and flavonoid contents in CBJ and CSJ, respectively. Antioxidant activity was significantly enhanced, particularly in DPPH (increased by 0.34 and 0.38 - fold), ABTS (increased by 2.76 and 1.40 - fold) scavenging activity, and FRAP (increased by 3.67 and 2.22 - fold). Widely targeted metabolomics identified 348 phenolic compounds, including butin, curcumin, demethoxycurcumin, they primarily enriched in flavonoid and anthocyanin biosynthesis pathways, and underwent TJ199 - mediated structural modifications (including hydroxylation, deglycosylation, and seven other reactions). Upregulated phenolic products (butin) exhibited stronger antioxidant activity than precursors (liquiritigenin) after biotransformation. These findings provided insights for developing corn bract and silk as food ingredients.

## Highlights

• Stains with antioxidant abilities were screened from acid porridge.
• *Lactobacillus. paracasei* TJ199 fermentation boosts FCBJ/FCSJ antioxidant activity.
• Phenolic compounds were key metabolites underlying antioxidant changes.
• Phenolic compounds were predominantly hydroxylated during fermentation.
• TJ199 fermentation elevated high-activity polyhydroxy phenolics abundance.

## Graphical abstract

**Figure:**
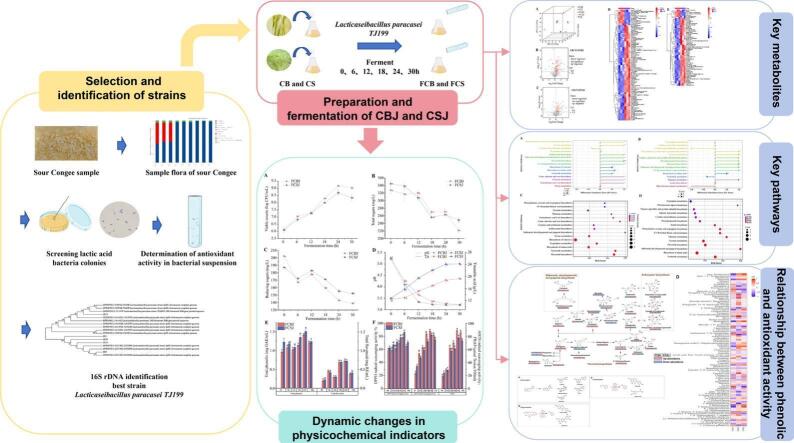

## 1 Introduction

Oxidative stress arises from an imbalance between the production of reactive oxygen species (ROS) and the body's antioxidant defenses (Li et al., 2024). This condition has been linked to the development of several chronic diseases, such as cardiovascular issues and neurodegenerative disorders (Jomova et al., 2024). In recent years, there has been growing interest in exploring natural antioxidants as potential therapeutic agents to mitigate oxidative stress-related damage. Among these, phenolic compounds have emerged as promising candidates due to their diverse biological activities and structural versatility (Prakoso, Babazadeh, & Wijayanti, 2023). The chemical structure of phenolic compounds largely influences their antioxidant capacity, particularly the number and position of hydroxyl groups on aromatic rings (Truong & Jeong, 2022). For instance, dihydroxylated caffeic acid exhibits superior antioxidant activity compared to its monohydroxylated counterpart, protocatechuic acid, highlighting the significance of structural modifications in enhancing biological activity (Gao, Li, Li, Zhang, & Liang, 2022).

Corn (*Zea mays L.*) is widely cultivated in regions such as Americas and Asia, with an annual production exceeding 1.2 billion metric tons, ranking as the second most produced grain globally and playing a critical role in economic development (Muddassir & Alotaibi, 2023). However, only 50 % of this production is utilized as food, while the remaining 50 % consists primarily of agricultural by-products such as corn silk and corn bract, which are currently underutilized as waste resources with a utilization rate of less than 10 % (Castillo-González, De Medina-Salas, Giraldi-Díaz, & Sánchez-Noguez, 2021). Corn silk and bract are rich in bioactive substances, notably, corn silk has been traditionally used in Chinese medicine as “longxu” (dragon's beard). In recent years, these by-products have garnered increasing attention due to their bioactive properties and functional potentials (Choudhary & Tahir, 2023). Studies have demonstrated that boiled juices derived from corn bract and silk possess hepatoprotective properties and may alleviate oxidative stress-induced damage (Lv et al., 2025). These positive effects are mainly due to their rich content of phenolic compounds, including polyhydroxylated formononetin, luteolin, and free delphinidin, which exhibit potent antioxidant activities (Li, Zhang, Deng, Zhang, & Li, 2022). However, a significant proportion (70 % - 80 %) of the phenolic compounds in these by-products exist in low-bioavailability forms, such as monohydroxy or non-hydroxylic group substitutions and glycoside conjugates. Moreover, conventional processing methods, particularly boiling, can lead to substantial loss of these bioactive compounds. These limitations highlight the necessity for innovative processing methods to improve the bioavailability and functionality of phenolic compounds in corn by - products.

During fermentation, lactic acid bacteria (LAB) utilize nutrients from the substrate for growth while facilitating the bioconversion of compounds through structural modifications, thereby increasing the content of metabolites with high antioxidant capacity (El Sheikha & Hu, 2020). For instance, fermentation of Kuntze juice by bifidobacteria has been shown to promote reactions such as deglycosylation and hydroxylation of flavonoids, resulting in enhanced antioxidant capacity (Wang et al., 2022).

Despite these advancements, research on the biotransformation of phenolic compounds in CBJ and CSJ remains limited. Previous studies on corn silk and corn bract have mostly focused on their antioxidant capacity and the classification of phenolic compounds. There are relatively few comprehensive studies on improving the bioavailability of phenolic compounds in plant-based foods through lactic acid bacteria fermentation. Thus, we herein identified a strain of L. *paracasei* TJ199 with high antioxidant activity and capable of growth and reproduction in CBJ and CSJ, a finding that may have significant implications for enhancing the polyphenol content in corn silk and corn bract. Using ultra-high-performance liquid chromatography-mass spectrometry (UHPLC-MS) - based widely targeted metabolomics, we investigated the changes in phenolic compound profiles before and after fermentation, with particular focus on the bioconversion pathways and their correlation with enhanced antioxidant activity. This research not only provides new insights intothe metabolic transformation of phenolic compounds but also offers a novel approach for value-added utilization of corn by-products.

## 2 Materials and methods

### 2.1 Materials

Corn bract and silk were obtained from an experimental farm at Jinzhou Medical University. Acid porridge samples were collected from three regions in China: Neimenggu (BTSZ), Guangxi (QSSZ), and Shanxi (YZSZ).

### 2.2 Screening for high-antioxidant activity strains

#### 2.2.1 Analysis of the dominant flora in acid porridge

Genomic DNA from BTSZ, QSSZ, and YZSZ samples were extracted and analyzed. PCR products from the same sample were mixed and then detected and quantified using 2 % agarose gel electrophoresis and fluorescence quantification technology (QuantiFluor™-ST Blue Fluorescence Quantification System). An Illumina library was constructed and Illumina sequencing was performed to determine the dominant flora.

#### 2.2.2 Preparation of complete bacterial suspension

The acidic porridge sample was mixed with normal saline in a 1:9 ratio for gradient dilution, then coated samples in an MRS solid medium at 37 °C for 48 h. In conjunction with cellular morphology, the strains exhibited positive gram staining and negative catalase activity at a concentration of 3 %. These strains were mixed with 25 % glycerin liquid and stored at −80 °C until use. Inoculate the obtained colonies into a fresh MRS medium and cultured at 37 °C for 24 h. Bacteria were collected through centrifugation, washed twice with sterile PBS, resuspended in PBS (Li et al., 2024).

#### 2.2.3 Hydroxyl radical scavenging ability of the strain

The determination of the strains' ability to scavenge hydroxyl radicals was slightly modified based on previous reports (Li, Gu, Yang, & Yu, 2023). A sequential and thorough mixing of 0.5 mL of the bacterial suspension, 0.5 mL FeSO_4_ (9 mmol/L), and 0.5 mL H_2_O_2_ (8.8 mmol/L) solutions, conducted and left for 10 min. Next, 0.5 mL of a 6 mmol/L salicylic acid solution was added and mixed, then incubated at 37 °C for 30 min.

#### 2.2.4 2,2-diphenyl-1-picrylhydrazyl (DPPH) and ferric reducing antioxidant power (FRAP) of the strain

DPPH radical scavenging activity was determined using the Jiang method with slight modifications (Jiang et al., 2021). Briefly, by dark reaction of 150 μL samples with 150 μL of DPPH-ethanol (0.2 mmol/L) solution for 30 min, absorbance was measured at 517 nm.

The method for determining FRAP was slightly modified based on Li's version (Li et al., 2021). After diluting the samples, 250 μL was combined with 150 μL of FRAP solution and reacted for 10 min. Absorbance was measured at 593 nm, the results were expressed as Trolox equivalents (μmol Trolox/100 mL).

#### 2.2.5 Identification of strains and homology analysis

The isolated strains were cultured to a stable level. The DNA fragment was amplified with a universal primer (27F, 5-AGAGTTTGATCCTGGCTCAG-3), the PCR products were sequenced by Shanghai Personal Biotechnology Co. Ltd. NCBI BLAST program to compare the concatenated sequence files with the NCBI 16S database, and MEGA software (version 9.0) was used to construct a phylogenetic tree.

### 2.3 Preparation and fermentation of CBJ and CSJ

Corn bract and corn silk were washed and dried, separately mixed them with distilled water at a ratio of 1:50 (*w*/w), then maintained in a gentle boiling state for 30 min. After filtering the juice, it was sterilized at 100 °C under high pressure for 10 min, to obtain CBJ and CSJ. Using corn bract and corn silk as substrates, we optimized the food-grade medium conducive to the growth and proliferation of TJ199 and adjusted the cell concentration to 8 Log CFU/mL. The activated strains (7 %, *v*/v) were cultured in sterilized CBJ and CSJ in 37 °C incubators, thus, the FCBJ and FCSJ were obtained. During the fermentation process. To explore the dynamic changes of the indicators during the fermentation process and determine the optimal fermentation time, samples were taken at six time points, namely 0 h, 6 h, 12 h, 18 h, 24 h and 30 h, for subsequent analysis.

### 2.4 Measurement of physicochemical properties

The plate count method was used to measure the viable cell count in the fermentation broth. The pH value of FCBJ and FCSJ samples were measured using a digital pH meter. Titrable acidity (TA) was measured through NaOH titration. Using glucose as the standard, the content of reducing sugars and total sugars was determined using the 5-dinitrosalicylic acid (DNS) and the phenol‑sulfuric acid method, respectively (Li et al., 2021).

### 2.5 The antioxidant capacity of the FCBJ and FCSJ

#### 2.5.1 DPPH radical scavenging assay and FRAP of the FCBJ and FCSJ

Same as 2.2.4.

#### 2.5.2 ABTS radical scavenging assay of the FCBJ and FCSJ

The assessment of ABTS radical scavenging capacity was performed based on the method of Rockenbach, with slight modifications (Rockenbach et al., 2011). The ABTS mixed solution was diluted with 80 % ethanol until the absorbance at 734 nm reached 0.70 ± 0.02. Then, 50 μL of diluted FCBJ and FCSJ samples were combined with 400 μL ABTS mixed solution, the absorbance was measured at 734 nm after the reaction time of 30 min. The results were expressed as Trolox equivalents (μmol Trolox/100 mL).

### 2.6 Measurement of total phenolic content (TPC) and total flavonoid content (TFC)

The TPC of the juice was measured using the Folin-Ciocalteu method (Pérez, Dominguez-López, & Lamuela-Raventós, 2023). 1 mL diluted samples were removed and mixed evenly with 0.5 mL folinol (10 %, *W*/*V*), 0.5 mL Na₂CO₃ (7.5 %, W/V), and reacted at 30 °C for 30 min. The absorbance of the samples was recorded at 765 nm in the dark, expressed as gallic acid equivalents (mg GAE/mL).

The TFC of the juice was measured using the aluminum chloride colorimetric method (Kwaw et al., 2018). 5 mL of diluted samples were added to 0.2 mL NaNO_2_ (50 %, *w*/*v*) reacted 10 min. Then that added 0.2 mL AlCl_3_ (100 %, w/v) reacted for 10 min. After that, 3 mL of NaOH (5 mol/L) was introduced, followed by the addition of 1.6 mL of 70 % ethanol, the absorbance of the samples were measured at absorbance of 510 nm after equilibration for 15 min, using rutin as the standard (mg RE/mL).

### 2.7 Widely targeted metabolomics analysis

#### 2.7.1 Metabolites extraction

200 μL of unfermented and fermented to 24 h CBJ and CSJ singles were transfered into Eppendorf tube and dried under nitrogen. Then, 400 μL of an internal standard solution, composed of methanol and water in a 3:1 ratio, which was added to the pre-cooled extraction liquid at −40 °C. The mixture were vortexed for 30 s and then sonicated for 15 min. Next, the samples were centrifuged (12,000 rpm, 15 min, 4 °C) and filtered using a 0.22 microporous membrane combined to prepare quality control (QC) samples.

#### 2.7.2 UHPLC- MS analysis

The UHPLC experiments were conducted using the EXION LC System (Sciex). The chromatographic separation of the target compounds was carried out using a Waters Acquity UPLC HSS T3 liquid chromatography column (2.1 × 100 mm, 1.8 μm). Mobile phase A contained 0.1 % formic acid in water, while mobile phase B was acetonitrile. The autosampler was maintained at a temperature of 4 °C, and a 2 μL injection volume was utilized with a A Sciex QTrap 6500+ (Sciex Technologies). The typical parameters for the ion source included an ion spray voltage of +5500/−4500 V, DP at ±100 V, temperature of 400 °C, Curtain Gas at 35 psi, Ion Source Gas 1:60 psi.

### 2.8 Statistical analysis

The original data encompassed 2 QC samples and 12 experimental samples, resulting in the extraction of 1053 peaks. MRM data collection and processing were carried out using SCIEX Analyst Software (version 1.6.3). The original MS data (.wiff) files were transformed into TXT format with MSConvert. Analysis of pathway was performed using KEGG and MetaboAnalyst.

All the experiments were carried out in triplicate. Statistical analyses were conducted using SPSS 25.0 (SPSS, Inc., USA), date was displayed as mean ± standard deviation (SD). Duncan's test was used for analysis of variance, when *P <* 0.05, the data were considered statistically significant.

## 3 Results

### 3.1 Screening and identification of lactic acid bacteria with high antioxidant activity

Acidic gruel is a type of food formed by natural fermentation of corn, resulting in a rich microbial community structure. The levels of *Lactobacillus* were highest in the acidic gruel samples (Fig. S1), with an average abundance of 79.83 %. Acidic gruel is a good source of LAB and a suitable substrate for screening high-quality strains.

Through comprehensive screening of 190 strains, we identified six candidates (X12, X20, N96, G199, G213, and G215) exhibiting both DPPH and hydroxyl radical-scavenging abilities exceeding 45 %, along with significant FRAP activity. The 16S rDNA amplicon sequencing results of the six strains submitted to the NCBI database for alignment analysis, facilitating the construction of a phylogenetic tree and enabling homology comparisons (Fig. S2). Among these, five strains showed highest similarity to *Lacticaseibacillus paracasei*, while one strain was closely related to *Furfurilactobacillus milii*. Among these, strain G199 demonstrated superior antioxidant properties, with DPPH and hydroxyl radical scavenging abilities of 60.92 ± 4.3 % and 54.81 ± 3.5 %, respectively, and FRAP value of 0.103 ± 0.01 μmol Trolox/mL. Further characterization confirmed strain G199 as L. *paracasei*, subsequently named L. *paracasei* TJ199.

### 3.2 Fermentation dynamics and enhancement of bioactive properties

**Fig. 1:**
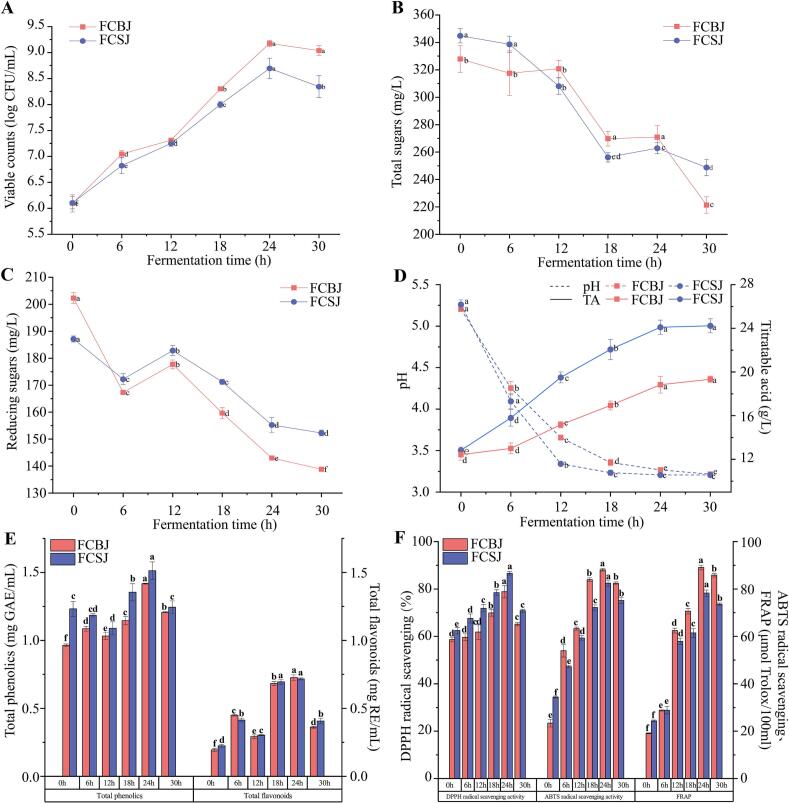
Viable cell counts of TJ199 (A), total sugars (B), reducing sugars (C), pH value and TA (D), total phenol and flavonoid content (E), antioxidant activity of CBJ and CSJ during fermentation (F). Significant differences were indicated by different letters (*p <* 0.05).

The fermentation kinetics of L. *paracasei* TJ199 in both FCBJ and FCSJ demonstrated robust microbial growth and metabolic activity (Fig. 1A). Viable cell counts reached their maximum at 24 h of fermentation, with 9.17 ± 0.06 and 8.69 ± 0.19 Log CFU/mL for FCBJ and FCSJ, respectively, indicating excellent adaptation of TJ199 to both substrates. The fermentation process significantly altered the physicochemical properties of the juices, with total sugar content decreasing by 32.5 % and 27.9 % in FCBJ and FCSJ, respectively, and reducing sugars showing reductions of 31.4 % and 18.6 % after 30 h of fermentation (*P* < 0.05) (Fig. 1B-C).

Concurrent with sugar consumption, we observed significant acidification of the fermentation media. The pH values decreased to 3.26 ± 0.02 and 3.21 ± 0.01 for FCBJ and FCSJ, respectively (*P* < 0.05), while TA increased to 19.33 ± 0.29 and 24.23 ± 0.64 mg/L (Fig. 1D). These changes in physicochemical parameters confirmed the metabolic activity of TJ199 and established optimal fermentation conditions for subsequent analyses.

The fermentation process significantly enhanced the bioactive profile of both juices. TPC reached maximum levels at 24 h of fermentation, with 1.51 ± 0.06 and 1.41 ± 0.003 mg GAE/mL for FCBJ and FCSJ, respectively, orresponding to 0.47 - fold and 0.23 - fold increases relative to CBJ and CSJ (*P* < 0.05) (Fig. 1E). Similarly, TFC showed substantial increases of 2.73-fold and 2.19-fold in FCBJ and FCSJ, respectively. The antioxidant capacity of the fermented juices showed remarkable improvement (Fig. 1F), with ABTS scavenging ability increasing by 2.76- and 1.40-fold, and FRAP values increasing by 3.67- and 2.22-fold in FCBJ and FCSJ, respectively (*P* < 0.05) (Fig. 1F). DPPH scavenging activity reached 86.6 % and 78.9 % for FCSJ and FCBJ, respectively, orresponding to 0.34 and 0.38 - fold increases relative to CBJ and CSJ, at the 24 h time point. Based on the above results, we decided to select 24 h as the optimal fermentation time and to use samples collected at this time for subsequent widely targeted metabolomics analysis.

### 3.3 Comprehensive analysis of metabolic profiles in fermented CBJ and CSJ

Widely targeted metabolomics analysis identified a total of 1053 metabolities spanning all sample groups (Fig. S3), with 1037 and 1047 metabolites in CBJ/FCBJ and CSJ/FCSJ, respectively. The detected metabolites encompassed diverse chemical classes, including alkaloids, flavones, phenols, phenylpropanoids, terpenes, coumarins, amino acids and their derivatives, and nucleotides and their derivatives. Notably, based on the carbon skeleton structure and the number and position of the substituents, 348 phenolic compounds were identified in both FCBJ and FCSJ, representing 33 % and 32 % of the total metabolites, respectively. Detailed phenolic compound profiles revealed distinct compositions: FCBJ contained 112 flavones, 92 polyphenols, 48 flavonoids, 44 phenylpropanoids, 30 coumarins, and 22 lignins (Table S1), while FCSJ comprised 112 flavones, 93 polyphenols, 47 flavonoids, 44 phenylpropanoids, 30 coumarins, and 22 lignans (Table S1). Among these phenolic compounds, there are 70 and 56 with VIP > 1 in the FCBJ and FCSJ groups, respectively, listed in Table S2 and Table S3 for subsequent metabolite analysis.

**Fig. 2:**
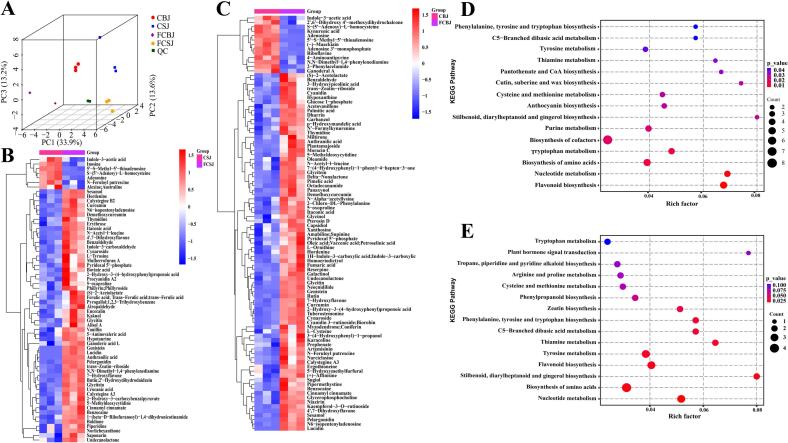
3 D scatter plot of PCA score plots of all metabolites (A). Classification heat map of total differential metabolites of CSJ VS FCSJ (B) and CBJ VS FCBJ (C), with colour indicating changes in concentration: red for upregulation and blue for downregulation. QC, quality control. PCA, Principal coordinate analysis. KEGG Enrichment for group CBJ VS FCBJ (D), CSJ VS FCSJ (E), the circles represent the ratio of differential metabolites to the total metabolites count in that pathway, the level of pathway enrichment is indicated by the size of the circles, larger circles indicate a higher level of enrichment. (For interpretation of the references to colour in this figure legend, the reader is referred to the web version of this article.)

Multivariate statistical analysis revealed significant metabolic alterations induced by TJ199 fermentation. Principal component analysis (PCA) demonstrated clear separation between fermented and unfermented samples (Fig. 2A), indicating substantial metabolic reprogramming. The analytical system exhibited excellent stability and reproducibility, as evidenced by quality control (QC) samples showing high correlation (0.95, > 0.7) and low relative standard deviation (RSD = 3.97 % for 2-chlorophenylalanine, < 20 %) (Fig. S4A—C). Orthogonal partial least squares-discriminant analysis (OPLS-DA) models further confirmed the metabolic differences, with robust model parameters (R^2^Y = 1, Q^2^ = 0.897 for CBJ/FCBJ; R^2^Y = 1, Q^2^ = 0.771 for CSJ/FCSJ) (Fig. S5A—B). These statistical parameters validate the reliability of our analytical approach and support subsequent differential metabolite analysis.

### 3.4 Differential metabolite analysis and functional annotation

Integrating OPLS-DA modeling with univariate and multivariate statistical analyses, we identified statistically significant differential metabolites (*P* < 0.05, VIP > 1) between unfermented and fermented CBJ and CSJ. The differential metabolites were systematically visualized using hierarchical clustering analysis (Fig. 2B-C), revealing distinct metabolic profiles between fermentation states.

Comparative analysis between CBJ and FCBJ identified 94 significantly altered metabolites, including 28 phenolic compounds that accounted for 30 % of the total differential metabolites. Among these phenolic compounds, 23 showed significant upregulation, including genistein, cyanidin, rutin, 4′,7-dihydroxyflavone, homoeriodictyol, curcumin, and glycinol, while (−)-maackiain was the only downregulated phenolic compound. Similarly, analysis of CSJ and FCSJ revealed 62 differential metabolites, with 22 phenolic compounds representing 35 % of the total. Notably, all of these phenolic compounds in CSJ/FCSJ comparison were upregulated, including genistein, 4′,7-dihydroxyflavone, butin, sesamol, vanillin, 7-hydroxyflavone and ferulic acid. Beyond phenolic compounds, other differential metabolites, including alkaloids, plant hormones, organic acids, amino acids and their derivatives showed consistent upregulation in both fermentation systems. In contrast, nucleotides and their derivatives were predominantly downregulated. This clustering pattern not only validated the reliability of our differential metabolite identification but also provided insights into the metabolites potentially influenced by TJ199 fermentation.

### 3.5 Metabolic pathway analysis and phenolic compound transformation

**Fig. 3:**
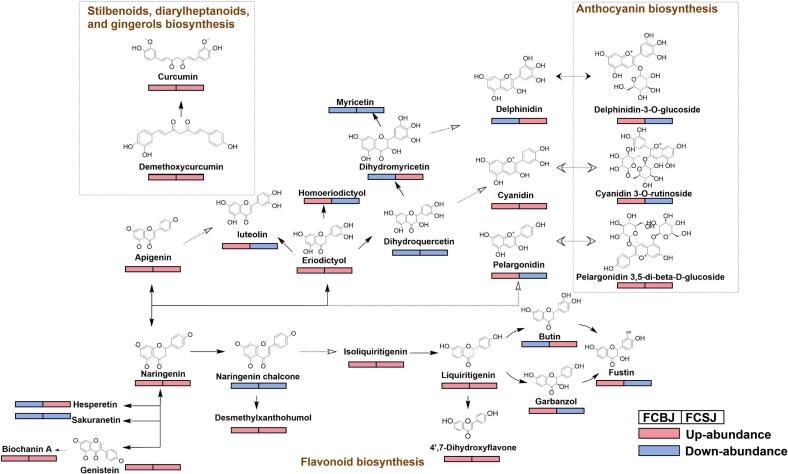
Metabolic changes of key metabolites in the three main metabolic pathways of fermented and unfermented CBJ and CSJ, solid lines were used to indicate direct conversions, while dotted lines were used to represent indirect conversions. The changes in compound abundance after fermentation are displayed by different colour, where red indicates upregulation and blue indicates downregulation. (For interpretation of the references to colour in this figure legend, the reader is referred to the web version of this article.)

KEGG pathway analysis revealed significant metabolic reprogramming following TJ199 fermentation, with 66 and 42 differential metabolic pathways identified in CBJ and CSJ, respectively. The top 15 significantly enriched pathways, ranked by rich factors, are presented in Fig. 2D-E. Based on stringent criteria (*P* < 0.05 and Rich Factor), we identified three primary metabolic pathways associated with phenolic compound biotransformation: (1) flavonoid biosynthesis, (2) anthocyanin biosynthesis, and (3) stilbenoid, diarylheptanoid, and gingerol biosynthesis. These pathways were selected for detailed investigation due to their central role in phenolic compound metabolism and transformation (Fig. 3).

**Fig. 4:**
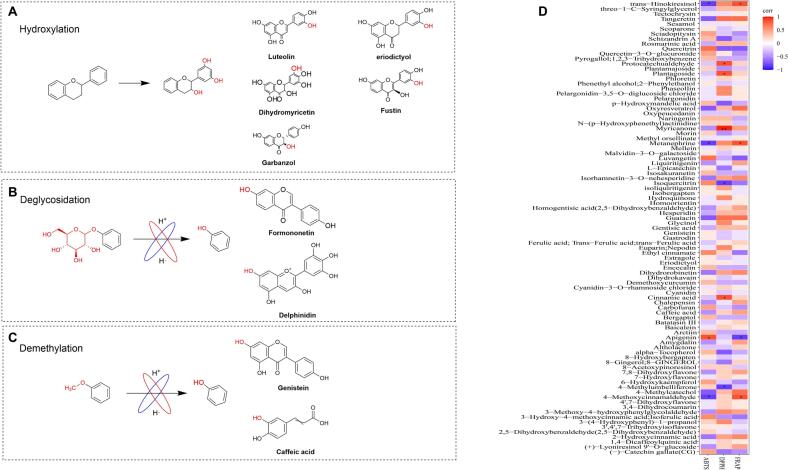
The mechanism of hydroxylation reaction and the key metabolites involved were described (A). The mechanism of deglycosylation reaction and the key metabolites involved (B). The mechanism of demethylation reaction and key metabolites involved (C), red indicates the functional groups that may undergo transformation. Pearson correlation coefficients of differential metabolites and antioxidant activities (D). (For interpretation of the references to colour in this figure legend, the reader is referred to the web version of this article.)

Comprehensive analysis of the KEGG database enabled systematic characterization of key phenolic compound potential biotransformation reactions in FCBJ and FCSJ (Table 1). We identified seven major types of structural modifications: hydroxylation, glycosylation, demethylation, redox, hydrolysis, hydrogenation, and isomerization. For each transformation type, we documented the specific precursors and products, their concentration trends in FCBJ and FCSJ, and the predicted enzymes involved in these biochemical reactions. Furthermore, three primary types of reactions were selected for analysis (Fig. 4A-C). This finding provides valuable insights into the metabolic engineering potential of TJ199 fermentation in modifying phenolic compound profiles.

**Table 1:** The primary reaction types, reactive substances, and predictive enzymes involved in fermented and unfermented CBJ and FCBJ.

Type	Precursors	FCBJ	FCSJ	Products	FCBJ	FCSJ	Predictive enzymes
Hydroxylation (9)	Apigenin	↑	↑	Luteolin	↑	↓	flavonoid 3′,5′-hydroxylase [EC:1.14.14.81]
	Liquiritigenin	↑	↑	Garbanzol	↑	↓	naringenin 3-dioxygenase [EC:1.14.11.9]
	Pinocembrin	↓	↓	Pinobanksin	↑	↓	naringenin 3-dioxygenase [EC:1.14.11.9]
	Taxifolin	↓	↓	Dihydromyricetin	↓	↓	flavonoid 3′,5′-hydroxylase [EC:1.14.14.81]
	Garbanzol	↑	↓	Fustin	↑	↑	flavonoid 3′-monooxygenase [EC:1.14.14.82]
	Liquiritigenin	↑	↑	Butin	↑	↓	flavonoid 3′-monooxygenase [EC:1.14.14.82]
	Naringenin	↑	↑	Eriodictyol	↑	↑	flavone synthase II [EC:1.14.19.76]
	4-Hydroxyphenylacetic acid	↑	↓	Homogentisic acid	↑	↑	4-hydroxyphenylacetate 1-monooxygenase[EC 1.14.13.18]
	4- Hydroxyphenylacetate	↑	↓	3,4-Dihydroxyphenylacetic acid	↑	↓	4-hydroxyphenylacetate 3-monooxygenase [EC:1.14.14.9]
Demethyla(5)	Prunetin	↑	↓	Genistein	↑	↑	isoflavone-7-*O*-methyltransferase [EC:2.1.1.150]
	Biochanin A	↑	↑	Genistein	↑	↑	2,7,4′-trihydroxyisoflavanone 4’-*O*-methyltransferase[EC:2.1.1.212 2.1.1.46]


Type	Precursors	FCBJ	FCSJ	Products	FCBJ	FCSJ	Predictive enzymes
	Sakuranetin	↓	↓	Naringenin	↑	↑	Naringenin 7-*O*-methyltransferase [EC:2.1.1.232]
	3,4-Dihydroxymandelic acid	↑	↓	4-Hydroxy-3-methoxymandelate	↑	↓	catechol *O*-methyltransferase [EC:2.1.1.6]
	Ferulic acid	↑	↑	Caffeic acid	↑	↓	caffeic acid 3-*O*-methyltransferase [EC:2.1.1.68 2.1.1.4]
Deglycosidation (5)	Formononetin 7-O-glucoside	↓	↓	Formononetin	↑	↓	isoflavone 7-O-glucosyltransferase [EC:2.4.1.170]
	Delphinidin-3-O-glucoside	↑	↓	Delphinidin	↓	↑	anthocyanidin 3-O-glucosyltransferase [EC:2.4.1.115]
	Rutin	↓	↓	Isoquercitrin	↑	↑	flavonol-3-O-glucoside L-rhamnosyltransferase [EC:2.4.1.159]
	Trifolirhizin	↓	↑	(−)-Maackiain	↓	↓	isoflavone 7-O-glucosyltransferase [EC:2.4.1.170]
	Coniferyl alcohol	↑	↓	Myzodendrone	↑	↓	coniferin beta-glucosidase[EC 3.2.1.126]
Oxidation (5)	Pinobanksin	↑	↓	Galangin	↓	↓	flavonol synthase [EC:1.14.20.6]
	Cyanidin	↑	↑	L-Epicatechin	↑	↑	anthocyanidin reductase [EC:1.3.1.77]
	Naringenin	↑	↑	Apigenin	↑	↑	flavone synthase II [EC:1.14.19.76]
	3,9-Dihydroxypterocarpan	↑	↓	Gycinol	↑	↑	3,9-dihydroxypterocarpan 6a-monooxygenase [EC:1.14.14.93]
	Dihydromyricetin	↓	↓	Myricetin	↓	↓	flavonol synthase [EC:1.14.20.6]


Type	Precursors	FCBJ	FCSJ	Products	FCBJ	FCSJ	Predictive enzymes
Hydrogenation (6)	Vanillin	↑	↑	Vanillic acid	↓	↓	vanillin dehydrogenase [EC:1.2.1.67]
	3-Methoxy-4-hydroxyphenylglycolaldehyde	↑	↑	3-Methoxy-4-hydroxymandelate	↑	↓	aldehyde dehydrogenase (NAD(P)+) [EC:1.2.1.5]


	Coniferylaldehyde	↑	↓	Coniferyl alcohol	↑	↓	cinnamyl-alcohol dehydrogenase [EC:1.1.1.195]
	Liquiritigenin	↑	↑	4′,7-Dihydroxyflavone	↑	↑	flavone synthase II [EC:1.14.19.76]
	Eriodictyol	↑	↓	Luteolin	↑	↓	flavone synthase I [EC:1.14.20.5]
	Phenylpropanoids	↑	↑	Cinnamic acid	↑	↓	[EC 1.14.13.14]
Isomerisation (4)	Metanephrine	↑	↑	3-Methoxy-4-hydroxyphenylglycolaldehyde	↑	↑	monoamine oxidase [EC:1.4.3.4]
	Isoliquiritigenin	↑	↑	Liquiritigenin	↑	↑	chalcone isomerase [EC:5.5.1.6]
	Homogentisate	↑	↑	2,5-Dihydroxybenzaldehyde	↑	↑	[EC:4.1.1.-]
	Naringenin chalcone	↓	↓	Naringenin	↑	↑	chalcone isomerase [EC:5.5.1.6]
Hydrolysis (1)	5-O-Caffeoylshikimic acid	↑	↓	Trans-caffeic acid	↑	↓	caffeoylshikimate esterase [EC:3.1.1.-]

Note: ↑ indicates that the metabolites were up-regulated following fermentation, ↓ indicates that the metabolites were down-regulated following fermentation.

### 3.6 Structure-activity relationship analysis of phenolic compounds

Pearson correlation analysis revealed significant structure-activity relationships between upregulated phenolic compounds and antioxidant capacities in fermented juices (Fig. 4D), the antioxidant potential of phenolic compounds is primarily determined by their chemical structure, particularly the nature and position of functional groups on the aglycone moiety. The DPPH radical-scavenging capacity showed a strong positive correlation with polyhydroxylated compounds, particularly myricanone (*P* < 0.01). In contrast, glycosylated compounds such as isoquercitrin exhibited significant negative correlations with DPPH scavenging activity (*P* < 0.05), suggesting that glycosylation may reduce antioxidant potential. Similarly, ABTS scavenging capacity demonstrated significant positive correlations with polyhydroxylated flavonoids like apigenin (*P* < 0.05), while compounds lacking hydroxyl substitutions, such as 4-methoxycinnamaldehyde, showed negative correlations (*P* < 0.05). These structure-activity relationships highlight the critical role of hydroxyl group positioning and glycosylation patterns in determining antioxidant efficacy. The strong positive correlations observed for polyhydroxylated compounds suggest that increased hydroxylation enhances electron donation capacity, thereby improving radical scavenging activity. Conversely, the negative correlations observed for glycosylated compounds may reflect steric hindrance effects or reduced bioavailability of the active aglycone moiety.

## 4 Discussion

### 4.1 Bacterial strains and fermentation characteristics

The antioxidant capacity of LAB has emerged as a critical research focus in recent years, particularly for their potential applications in functional food development (Phupaboon et al., 2024). Our findings demonstrate that L. *paracasei* TJ199 exhibits superior antioxidant properties compared to previously characterized LAB strains. Notably, TJ199's DPPH scavenging ability (60.92 %) significantly exceeds the 30 % threshold typically associated with high-antioxidant capacity (Abduxukur, Tursuntay, Zhu, Wang, & Rahman, 2023), while its hydroxyl radical-scavenging capacity (50.48 %) surpasses that of the well-characterized probiotic strain L. *rhamnosus* GG (30.5 %) and is comparable to high-performance LAB strains isolated from traditional fermented products (Shi et al., 2019).

The fermentation performance of TJ199 in corn-based substrates was particularly noteworthy. Without nutritional supplementation, viable cell counts exceeded 8 log CFU/mL in both FCBJ and FCSJ, meeting commercial standards for probiotic products (Kaprasob, Kerdchoechuen, Laohakunjit, Sarkar, & Shetty, 2017). This robust growth can be attributed to TJ199's origin from corn - associated materials and the rich nutritional profile of CBJ and CSJ, which provide an optimal fermentation substrate. The observed metabolic dynamics, including rapid sugar consumption and transient increases in sugar content, are consistent with previous reports on LAB fermentation of plant-based materials (Zheng et al., 2023). These phenomena may be explained by TJ199's production of exopolysaccharides during exponential growth, which could facilitate microbial proliferation and substrate utilization (Susalam, Harnentis, Marlida, Jamsari, & Ardani, 2024).

The fermentation process induced significant acidification, this acidification may driven by the conversion of sugars to organic acids (as evidenced by increased levels of citric acid and azelaic acid in mass spectrometry analysis), created an optimal environment for TJ199 growth while inhibiting potential contaminants (Yang et al., 2024). Despite using the same strain, we observed notable differences in the physicochemical properties of FCBJ and FCSJ, suggesting substrate-specific metabolic adaptations and biotransformation patterns in TJ199.

### 4.2 Biotransformation of phenolic compounds and enhancement of antioxidant activity

The fermentation process significantly enhanced the antioxidant capacity, TPC, TFC of both FCBJ and FCSJ. Mass spectrometry analyses revealed a notable upregulation of polyhydroxylated and free phenolic compounds, suggesting that L. *paracasei* TJ199 fermentation promotes the synthesis or release of bioactive flavonoids and phenols. KEGG pathway analysis indicated that this biotransformation is likely mediated by the activation of key enzymes, such as hydroxyl transferases and glycosyltransferases, which facilitate the conversion of phenolic precursors into more bioactive forms (Cui, Zhang, Pan, Hu, & Wu, 2024). These findings underscore the importance of metabolic pathway analysis in understanding the biotransformation mechanisms of phenolic compounds during fermentation.

In the flavonoid biosynthesis pathway, liquiritigenin emerged as a key intermediate, interacting with five metabolites (Fig. 3). In FCSJ, the abundance of butin was up-regulated, it was likely that liquiritigenin was hydroxylated to form butin, which is a compound with superior antioxidant activity that mitigates DNA oxidative damage and protects mitochondria under stress conditions. This transformation may catalyzed by flavonoid 3′-monooxygenase [EC:1.14.14.82] (Table 1). Similarly, in FCBJ, the concentrations of 4′,7-dihydroxyflavone and garbanzol were significantly elevated after fermentation. Both compounds exhibit potent antioxidant properties, with 4′,7-dihydroxyflavone also demonstrating antitumor activity and enhanced transport protein binding, likely due to its polyhydroxyl structure (Jiang, Su, Liu, Zheng, & Li, 2020). Naringenin, another critical phenolic compound in the flavonoid biosynthesis pathway, was converted into homoeriodictyol, myricetin, pelargonidin, and apigenin (Fig. 3). These upregulated compounds showed strong antioxidant activities, with apigenin exhibiting a significant positive correlation with ABTS scavenging capacity and FRAP (*P* < 0.05) (Fig. 4D). Myricetin, derived from the hydroxylation of naringenin, demonstrated even greater antioxidant activity than its precursor (Pu, Deng, Chen, Yang, & Liang, 2023).

In the anthocyanin biosynthesis pathway, cyanidin was significantly upregulated in FCBJ, while its glycosylated form, cyanidin 3-O-rutinoside, decreased. This transformation, likely catalyzed by anthocyanidin 3-O-glucosyltransferase [EC:2.4.1.115], involves the deglycosylation of cyanidin 3-O-rutinoside to yield cyanidin (Fig. 3). Cyanidin exhibits stronger antioxidant and bioactive properties than its glycosylated precursor, contributing to the enhanced antioxidant activity of FCBJ. Similarly, the abundance of pelargonidin increased significantly in FCSJ, potentially due to the deglycosylation of pelargonidin-3,5-O-diglucoside chloride. Pelargonidin is known for its potent antioxidant and anti-inflammatory activities (Singh, Singh, Kaur, & Singh, 2018).

The stilbenoid, diarylheptanoid, and gingerol biosynthesis pathways showed fewer metabolites and transformations compared to the flavonoid and anthocyanin pathways (Fig. 3). However, metabolic data analysis revealed significant increases in the concentrations of curcumin and demethoxycurcumin in FCBJ and FCSJ, despite the downregulation of chlorogenic acid. These metabolites are known for their strong antioxidant and biological activities (Fu et al., 2022), making them promising candidates for functional foods and pharmaceuticals. The upregulation of these high-antioxidant phenolic compounds likely explains the enhanced antioxidant activity observed in FCBJ and FCSJ.

### 4.3 Major transformation types and their impact on antioxidant activity

Based on the three main metabolic pathways enriched in phenolic compounds, we comprehensively analyzed the biotransformation of phenolic compounds following TJ199 fermentation. The structural modifications of phenolic compounds, particularly the presence and position of key functional groups, play a crucial role in determining their antioxidant capacity. Previous studies have demonstrated that modifications such as hydroxylation, demethylation, and deglycosylation can significantly alter the metabolic characteristics and biological activities of phenolic compounds (Crascì, Lauro, Puglisi, & Panico, 2018). Using metabolomic data and the KEGG database, we identified two primary structural modifications in FCBJ and FCSJ: (1) hydroxylation reactions, which increase the number of key functional groups, and (2) hydrolysis of glycosidic or hydrogen bonds, which converts bound phenols into highly bioactive forms.Table 1 summarizes the specific reactions, metabolites, predicted enzymes.

#### 4.3.1 Hydroxylation reactions

Mass spectrometry and KEGG database analysis revealed nine hydroxylation reactions in FCBJ and FCSJ, with seven of the resulting products being flavonoids (Table 1). Most hydroxylated metabolites exhibited higher antioxidant activity than their precursors. For example, luteolin, fustin, and eriodictyol, which contain additional hydroxyl groups on the B-ring, demonstrated significantly greater antioxidant capacity compared to their low-hydroxyl or non-hydroxyl precursors, such as apigenin, garbanzol, and naringenin (Kongpichitchoke, Hsu, & Huang, 2015).

The antioxidant activity of these hydroxylated compounds is enhanced compared to their precursors, likely due to the number and specific positioning of hydroxyl groups on the aromatic rings. Specifically, luteolin, fustin, and eriodictyol have one additional hydroxyl substituent on the B-ring compared with their precursors, resulting in a structure with two hydroxyl groups (Fig. 3). Previous reports have shown that the presence of two or more hydroxyl groups on the B-ring was a critical feature for flavonoids to exhibit biological activities, such as free radical scavenging (Alseekh, de Souza, Benina, & Fernie, 2020). In addition, luteolin and dihydromyricetin, with hydroxyl substitutions at positions 3 and 5, form 3′,4′-dihydroxy and 3′,4′,5′-trihydroxy structures, respectively (Fig. 4A). These structural features are critical for free radical scavenging and other biological activities, the more hydroxyl substitutions present at sites 3, 4, and 5 on the B-ring of flavonoids, the stronger their inhibitory capacity against oxidases and overall antioxidant activity.

Despite the growing interest in hydroxylation reactions, the precise mechanisms underlying their impact on biological activity remain poorly understood. Additional studies are required to clarify the structural changes and their functional effects in phenolic transformations.

#### 4.3.2 Deglycosylation and demethylation reactions

We identified five deglycosylation and five demethylation reactions during the transformation of phenolic compounds in FCBJ and FCSJ (Table 1). These reactions typically involve the hydrolysis of glycosidic or methyl bonds, converting bound phenols into stronger antioxidant forms with increased hydroxyl substitutions. For example, the increased concentrations of formononetin and delphinidin were likely due to the enzymatic activity of isoflavone 7-O-glucosyltransferase [EC:2.4.1.170] and anthocyanidin 3-O-glucosyltransferase [EC:2.4.1.115], which catalyze the release of these compounds from their glycosidic forms (Table 1). Both formononetin and delphinidin exhibited stronger antioxidant activity than their glycosylated precursors (Gocer et al., 2016), likely due to the conversion of glycosyl groups into hydroxyl groups at key positions, including site 7 and 3 (Fig. 4B). This process may also involve sequential proton loss electron transfer (SPLET), enhancing free radical scavenging capacity (Curiel & Landete, 2022). The glycosides released during deglycosylation may also serve as carbon sources for LAB, potentially promoting microbial growth and further enhancing fermentation efficiency.

Demethylation reactions further contributed to the enhanced antioxidant activity of FCBJ and FCSJ. For instance, the increased levels of genistein and caffeic acid likely resulted from the demethylation of prunetin and ferulic acid, respectively (Table 1). The replacement of methyl groups with hydroxyl groups at position 7 on the A-ring (genistein) and position 3 on the B-ring (caffeic acid) increased the number of hydroxyl substitutions, thereby enhancing antioxidant activity (Fig. 4C). Additionally, electronic transfer during demethylation may generate reactive species (O• and O−), further increasing biological activity (Chen et al., 2018).

#### 4.3.3 Other transformation reactions

In addition to hydroxylation, deglycosylation, and demethylation, phenolic compounds in FCBJ and FCSJ underwent various other structural transformations, including redox, hydrogenation, hydrolysis, dehydration, and isomerization reactions. For example, hydrogenation reactions converted vanillin and liquiritigenin into vanillic acid and 7,4′-dihydroxyflavone, respectively, both of which exhibited stronger antioxidant and biological activities than their precursors (Watson et al., 2015). These transformations highlight the diverse metabolic capabilities of TJ199 and its potential for producing bioactive compounds.

In summary, our investigation of the metabolic pathways, biotransformations, and functional activities of phenolic compounds in FCBJ and FCSJ provides valuable insights for the development of functional foods from corn by - products. However, the specific enzymes and mechanisms underlying these transformations remain largely unexplored. Future studies should aim to clarify these mechanisms by employing advanced metabolomic and localization technologies. Additionally, the structure-activity relationships observed in this study, consistent with findings from other fermented products such as jujube puree (Li et al., 2022), underscore the need for further exploration of phenolic compound activities in vivo.

## 5 Conclusions

This study demonstrates that L. *paracasei* TJ199 fermentation significantly enhances the antioxidant properties of corn bract and silk juices through biotransformation of phenolic compounds. The identified 348 phenolic compounds, primarily enriched in flavonoid and anthocyanin biosynthesis pathways, undergo structural modifications including hydroxylation, deglycosylation, and demethylation, resulting in enhanced antioxidant activity. However, the relationship between the specific biotransformation mechanisms in the reaction and the antioxidant activity remains to be studied. Therefore, in future research, we will investigate the relationship between the metabolism of specific phenolic compounds after lactic acid bacteria fermentation and their antioxidant activity, providing a strong theoretical basis for improving the bioactivity of corn silk and corn bract. These findings provide valuable insights for the development of functional foods from corn by-products and highlight the potential of LAB fermentation in improving the bioavailability of phenolic compounds.

## CRediT authorship contribution statement

**Xiaohui Tang:** Writing – original draft, Software, Methodology, Data curation, Conceptualization. **Huanyong Lv:** Methodology, Investigation. **Hailong Wang:** Formal analysis. **Huixin Yang:** Software, Resources. **Baogang Zhang:** Resources. **Ling Cong:** Software. **Xinyi Li:** Methodology. **Menghan Ma:** Methodology. **Yunhe Xu:** Writing – review & editing, Supervision, Project administration, Funding acquisition. **Lili Zhang:** Writing – review & editing, Supervision, Project administration, Funding acquisition.

## Declaration of competing interest

The authors declare that they have no known competing financial interests or personal relationships that could have appeared to influence the work reported in this paper.

## Data Availability

Data will be made available on request.

## Appendix A Supplementary data

Supplementary material 1 Image 1

Supplementary material 2 Image 2
