# Supplementary material for: Enhancing antioxidant activity of corn bract and silk juices through biotransformation of polyphenols by Lactobacillus paracasei TJ199 fermentation
Source: Food Chem X. 2025 May 26;28:102586. doi: 10.1016/j.fochx.2025.102586 (PMC12159919; doi:10.1016/j.fochx.2025.102586)
Supplement: Supplementary file 2 — Supplementary material 2 [file mmc2.docx]

Fig. S1. The variation in the composition of dominant species among different acidic gruel samples.

Fig. S2. Construction of phylogenetic tree of six strains.

Fig. S3. The composition and classification of the top 20 metabolites by proportion before and after fermentation in the CBJ and CSJ groups.

Fig. S4. QC sample extraction ion flow diagram (A). TIC diagram of all QC samples (B). EIC diagram of internal standard 2-chlorophenylalanine in QC sample (C).

Fig. S5. The OPLS-DA models for the CBJ and FCBJ, CSJ and FCSJ.

Table S2. Phenolic compounds with VIP > 1 in the CBJ and FCBJ.

Table S3. Phenolic compounds with VIP > 1 in the CSJ and FCSJ


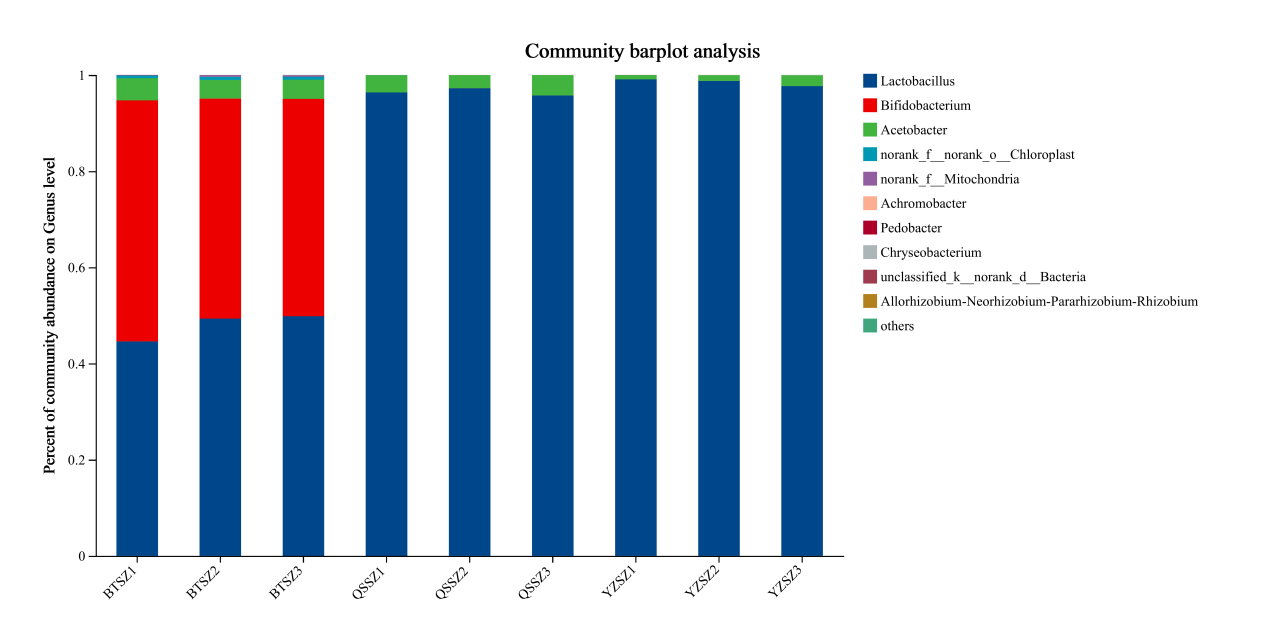


Fig. S1. The variation in the composition of dominant species among different acidic gruel samples.The x-axis representing sample names and the y-axis representing the proportion of each species in that sample. Different colored bars represent different species, and the height of the bars indicates the size of the proportion for each species.


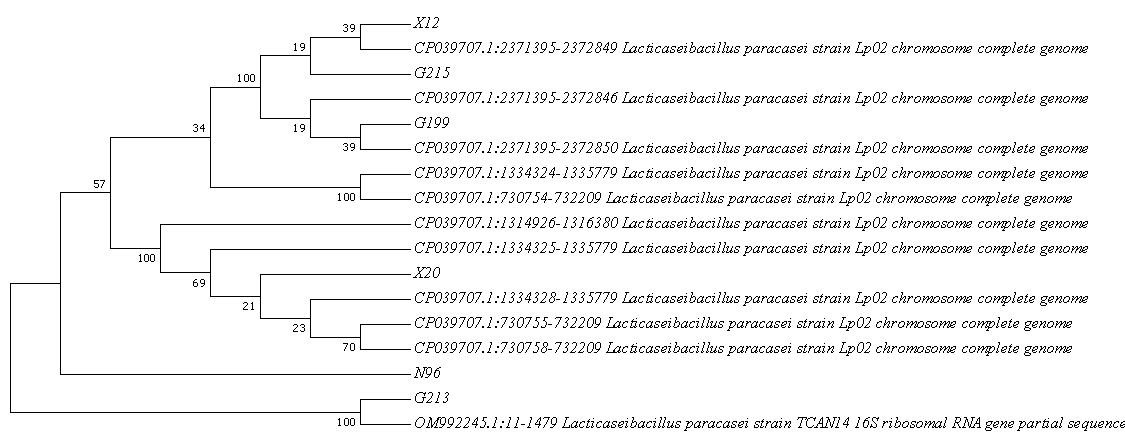


Fig. S2. Construction of phylogenetic tree of six strains


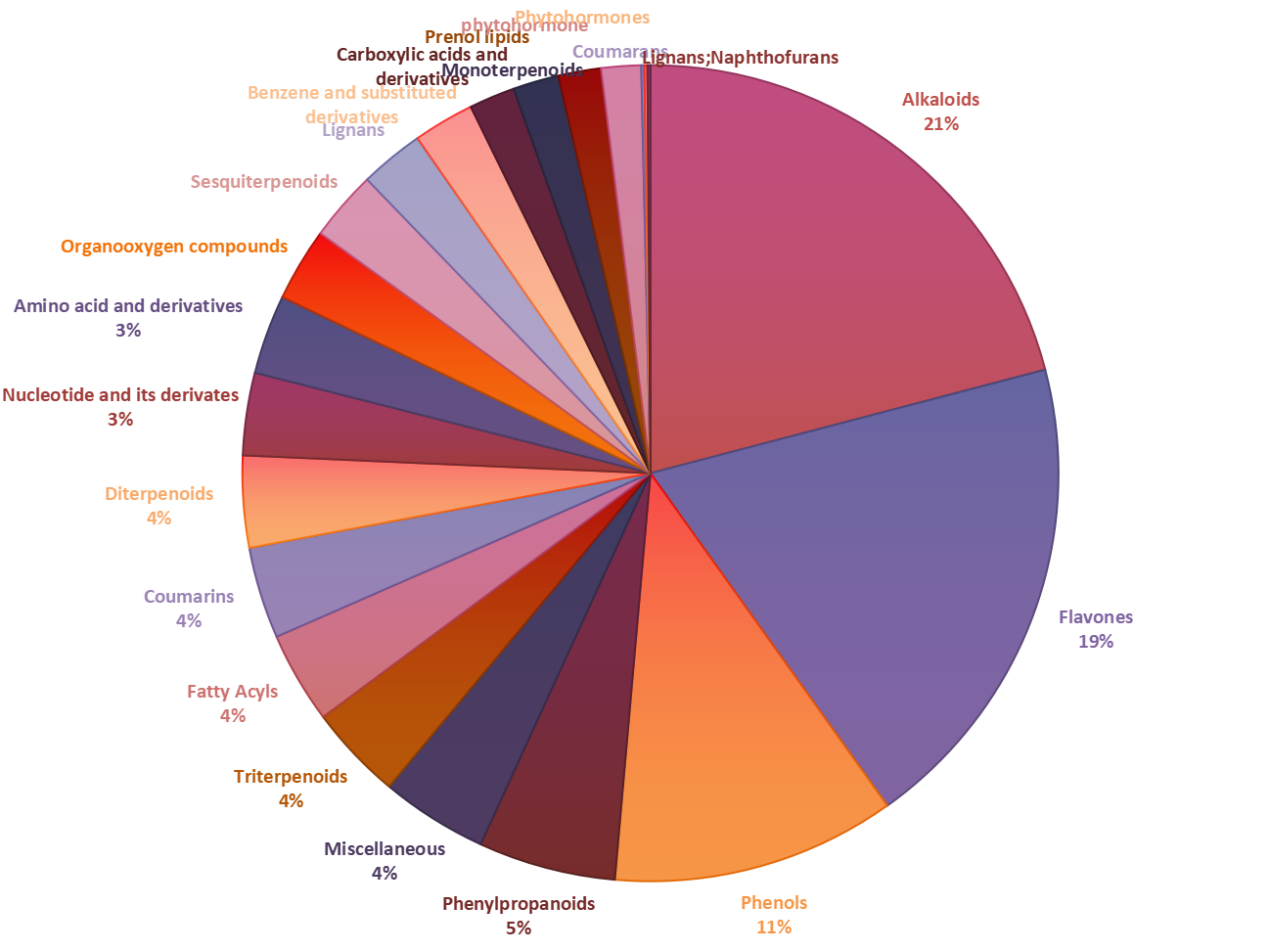


Fig. S3. The composition and classification of the top 20 metabolites by proportion before and after fermentation in the CBJ and CSJ groups.


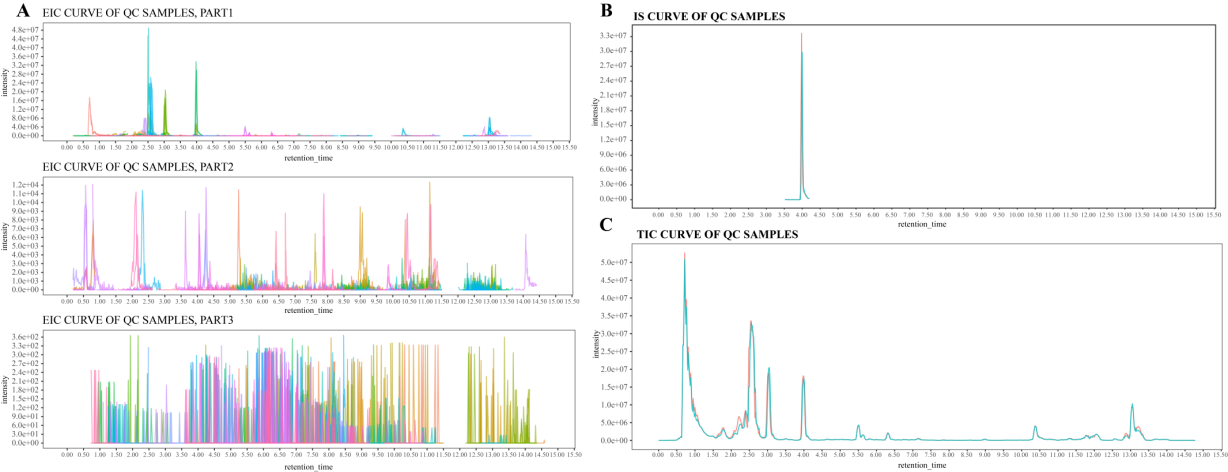


Fig. S4. QC sample extraction ion flow diagram (A). TIC diagram of all QC samples(B). EIC diagram of internal standard 2-chlorophenylalanine in QC sample (C).


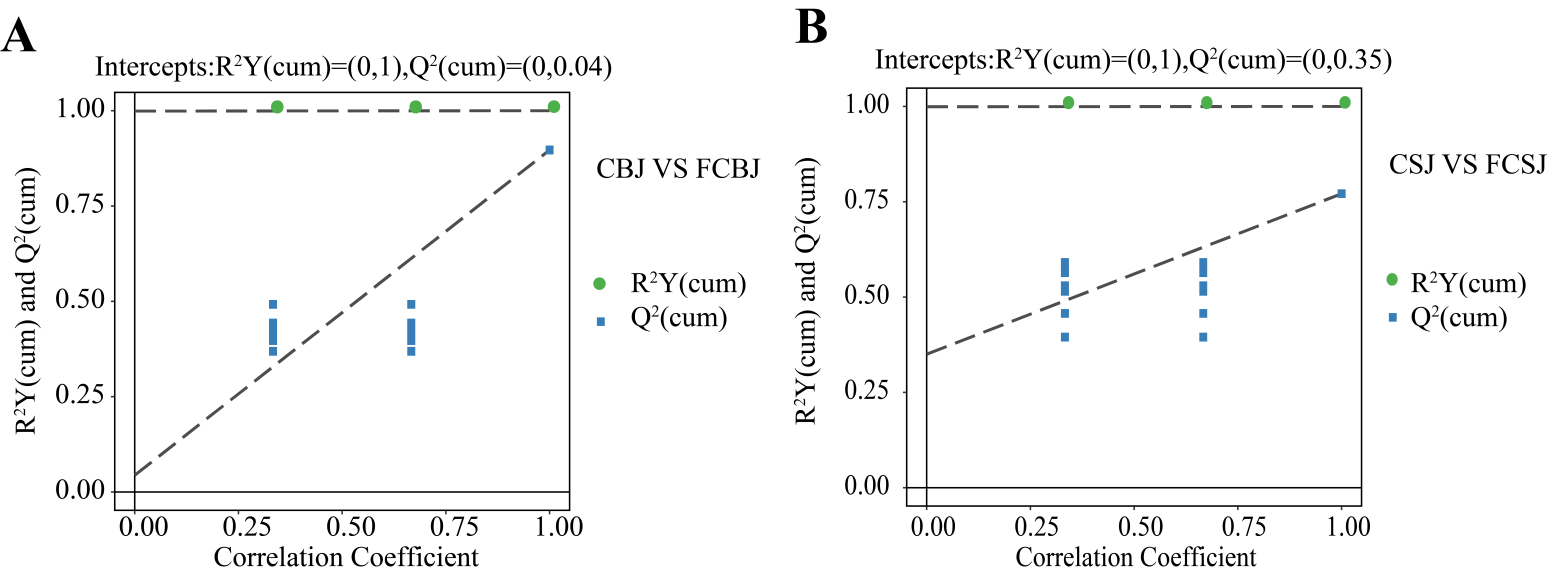


Fig. S5. The OPLS-DA models for the CBJ and FCBJ (A), CSJ and FCSJ (B).

Table S2. Phenolic compounds with VIP > 1 in the CBJ and FCBJ

| Class | Compound name | VIP | Class | Compound name | VIP |
| --- | --- | --- | --- | --- | --- |
| Flavonoids (21) | Cyanidin | 1.62 | Phenols (17) | Cynaroside | 1.74 |
|  | Pelargonidin | 1.75 |  | Kaempferol-3-O-rutinoside | 1.74 |
|  | Pinostrobin | 1.15 |  | Diosmin | 1.47 |
|  | Glycinol | 1.31 |  | Myzodendrone |  |
|  | Garbanzol | 1.59 |  | 4-Methylcatechol | 1.37 |
|  | 6-Hydroxykaempferol | 1.18 |  | Acetovanillone | 1.73 |
|  | Daidzein | 1.28 |  | Pyrogallol;1,2,3-Trihydroxybenzene | 1.02 |
|  | 4',7-Dihydroxyflavone | 1.75 |  | Sesamol | 1.19 |
|  | 7-Hydroxyflavone | 1.59 |  | Demethoxycurcumin | 1.74 |
|  | Vicenin 2 | 1.16 |  | Moracin C | 1.74 |
|  | Pelargonidin-3,5-O-diglucoside chloride | 1.09 |  | Octyl Gallate | 1.74 |
|  | Quercetin-3-O-sophoroside | 1.38 |  | Dihydrokavain | 1.47 |
|  | Quercetin-7-O-beta-D-glucopyranoside | 1.01 |  | 4-(Ethoxymethyl)phenol | 1.75 |
|  | Isoquercitrin | 1.36 |  | Curcumin | 1.50 |
|  | Liquiritigenin | 1.18 |  | Benzaldehyde | 1.39 |
|  | 2,3-Dehydrosilybin A | 1.21 |  | Vanillic acid | 1.50 |
|  | Buddlenoid A | 1.22 |  | p-Hydroxymandelic acid | 1.50 |
|  | Prunetin | 1.34 |  | 7-(4-Hydroxyphenyl)-1-phenyl-4-hepten-3-one | 1.71 |
|  | (-)-Maackiain | 1.73 |  | Cardanol | 1.74 |
|  | Homoeriodictyol | 1.64 |  | Niazirin | 1.67 |
|  | Cyanidin 3-rutinoside | 1.73 |  | 4'-O-Methylirenolone | 1.17 |
| Flavones (12) | Spinosin | 1.05 | Phenylpropanoids (12) | 3,4-Dimethoxycinnamic acid | 1.32 |
|  | Genistein | 1.76 |  | 1-Caffeoylquinic acid | 1.38 |
|  | Diosmetin | 1.37 |  | Ferulic acid; | 1.27 |
|  | Glycitein | 1.73 |  | 3-Hydroxy-4-methoxycinnamic acid | 1.32 |
|  | Protocatechualdehyde | 1.02 |  | Trans-caffeic acid | 1.20 |
|  | Baicalin | 1.19 |  | 3-(4-Hydroxyphenyl)-1-propanol | 1.52 |
|  | Glycitin | 1.74 |  | Lithospermic acid | 1.07 |

Table S2 (continued )

| Class | Compound name | VIP | Class | Compound name | VIP |
| --- | --- | --- | --- | --- | --- |
| Phenylpropanoids | Phillyrin | 1.20 | Coumarins | 3-Hydroxycoumarin | 1.04 |
|  | DL-Benzylsuccinic acid | 1.31 |  | Luvangetin | 1.36 |
|  | Plantamajoside | 1.69 | Lignans (4) | Arctiin | 1.13 |
|  | Cinnamyl cinnamate | 1.63 |  | Pinoresinol dimethyl ether | 1.05 |
|  | Ethyl cinnamate | 1.42 |  | Veraguensin | 1.36 |
| Coumarins (4) | Isobergapten | 1.40 |  | Schizandrin A | 1.32 |
|  | Alloimperatorin | 1.05 |  |  |  |

Table S3. Phenolic compounds with VIP > 1 in the CSJ and FCSJ

| Class | Compound name | VIP | Class | Compound name | VIP |
| --- | --- | --- | --- | --- | --- |
| Flavonoids (17) | Butin | 1.92 | Phenols | Sesamol | 1.90 |
|  | Malvidin 3-glucoside | 1.36 |  | Demethoxycurcumin | 1.81 |
|  | Flavone | 1.19 |  | Methyl benzoate | 1.62 |
|  | Pelargonidin | 1.89 |  | Encecalin | 1.67 |
|  | Baicalein | 1.52 |  | Altholactone | 1.11 |
|  | 4',7-Dihydroxyflavone | 1.88 |  | Dihydrokavain | 1.42 |
|  | 7-Hydroxyflavone | 1.94 |  | Kakuol | 1.68 |
|  | Vicenin 2 | 1.23 |  | 4-(Ethoxymethyl)phenol | 1.30 |
|  | Saponarin | 1.47 |  | Vanillin | 1.68 |
|  | Lonicerin | 1.22 |  | Curcumin | 1.92 |
|  | Isoscoparin | 1.37 |  | Benzaldehyde | 1.75 |
|  | Peonidin-3-glucoside | 1.12 |  | Mulberrofuran A | 1.68 |
|  | Malvidin-3-O-galactoside | 1.14 |  | p-Hydroxymandelic acid | 1.08 |
|  | Dihydrorobinetin | 1.18 |  | alpha-Hexylcinnamaldehyde | 1.60 |
|  | Procyanidin A2 | 1.69 | Phenylpropanoids (8) | 3,4-Dimethoxycinnamic acid | 1.28 |
|  | Tectochrysin | 1.18 |  | Chlorogenic acid | 1.16 |
|  | (-)-Maackiain | 1.24 |  | 4-Methoxycinnamaldehyde | 1.04 |
| Flavones (10) | Spinosin | 1.17 |  | Ferulic acid | 1.66 |
|  | Genistein | 1.94 |  | Benzyl cinnamate | 1.05 |
|  | Diosmetin | 1.26 |  | Dihydroconiferyl alcohol | 1.10 |
|  | Glycitein | 1.91 |  | Phillyrin | 1.59 |
|  | Protocatechualdehyde | 1.51 |  | Cinnamyl cinnamate | 1.86 |
|  | Myricetin | 1.54 | Coumarins (3) | Esculetin | 1.38 |
|  | Glabridin | 1.24 |  | Decursinol | 1.38 |
|  | Glycitin | 1.85 |  | Scoparone | 1.14 |
|  | Cynaroside | 1.84 | Lignans (3) | Guaiacin | 1.30 |
|  | Icariin | 1.26 |  | 8-Hydroxypinoresinol | 1.31 |
| Phenols (15) | Pyrogallol | 1.74 |  | Podophyllotoxinone | 1.14 |
